# Predictors of nonunion and reoperation in patients with fractures of the tibia: an observational study

**DOI:** 10.1186/1471-2474-14-103

**Published:** 2013-03-22

**Authors:** Katie Fong, Victoria Truong, Clary J Foote, Brad Petrisor, Dale Williams, Bill Ristevski, Sheila Sprague, Mohit Bhandari

**Affiliations:** 1 Division of Orthopaedic Surgery, McMaster University, Hamilton, Canada; 2 Department of Clinical Epidemiology and Biostatistics, McMaster University, Hamilton, Canada

**Keywords:** Tibial shaft fractures, Reoperation, Secondary intervention, Fracture prognostic index, Fracture characteristics, Nonunion, Cortical continuity

## Abstract

**Background:**

Tibial shaft fractures are the most common long bone fracture and are prone to complications such as nonunion requiring reoperations to promote fracture healing. We aimed to determine the fracture characteristics associated with tibial fracture nonunion, and their predictive value on the need for reoperation. We further aimed to evaluate the predictive value of a previously-developed prognostic index of three fracture characteristics on nonunion and reoperation rate.

**Methods:**

We conducted an observational study and developed a risk factor list from previous literature and key informants in the field of orthopaedic surgery, as well as via a sample-to-redundancy strategy. We evaluated 22 potential risk factors for the development of tibial fracture nonunion in 200 tibial fractures. We also evaluated the predictive value of a previously-identified prognostic risk index on secondary intervention and/or reoperation rate. Two individuals independently extracted the data from 200 patient electronic medical records. An independent reviewer assessed the initial x-ray, the post-operative x-ray, and all available sequential x-rays. Regression and chi-square analysis was used to evaluate potential associations.

**Results:**

In our cohort of patients, 37 (18.5%) had a nonunion and 27 (13.5%) underwent a reoperation. Patients with a nonunion were 97 times (95% CI 25.8-366.5) more likely to have a reoperation. Multivariable logistic regression revealed that fractures with less than 25% cortical continuity were predictive of nonunion (odds ratio = 4.72; p = 0.02). Such fractures also accounted for all of the reoperations identified in our sample. Furthermore, our data provided preliminary validation of a previous risk index predictive of reoperation that includes the presence of a fracture gap post-fixation, open fracture, and transverse fracture type as variables, with an aggregate of fracture gap and an open fracture yielding patients with the highest risk of developing a nonunion.

**Conclusions:**

We identified a significant association between degree of cortical continuity and the development of a nonunion and risk for reoperation in tibial shaft fractures. In addition, our study supports the predictive value of a previous prognostic index, which inform discussion of prognosis following operative management of tibial fractures.

## Background

Tibial shaft fractures are the most commonly-occurring long bone fracture and are prone to a number of complications, many of which may require additional treatment in the form of secondary therapies or revision surgery to promote bony healing. Orthopaedic injuries represent 67% of injury admissions to Canadian hospitals [1]. Fractures and dislocations of the lower limb represent 38% of all injury admissions with a total of nearly 86,000 injury admissions due to fractures [1]. It is estimated that by 2020, disability from traffic accidents (the major cause of fractures) will rank in the top three of all causes of disability [2].

Complications in fracture healing such as delayed union or nonunion occur in 4% to 48% of tibial shaft fractures, often resulting in the need for secondary intervention or additional treatment to stimulate bony union [3]. Reoperations are an example of such secondary intervention which can, and often does, result in a considerable impact in patient function and quality of life. Currently, there is much uncertainty with regards to what fracture characteristics predict fracture complications such as nonunion, particularly such cases which call for the need for secondary interventions in tibial shaft fracture patients [4]. Clinical and experimental studies have identified a number of potential factors that may help to predict such fracture complications [5-24]. However, these studies are often limited by single surgeon experiences, the use of outcome measures that are subject to variable interpretation, and limited rationale for the choice of prognostic variables. In addition, subsequent validation of previously developed prognostic indices for fracture healing is required [25].

Evidence is far from conclusive on what factors are predictive of fracture complications. To address these issues, we conducted a retrospective observational study among patients with operatively-managed fractures of the tibial shaft with the following key objectives: (1) to determine what fracture characteristics at the time of initial injury (baseline) are predictive of nonunion and the need for reoperation, (2) to provide preliminary validation of a previously-developed fracture prognostic index, and (3) to determine if certain aggregates of the variables included in the prognostic index have a higher predictive value for nonunion and reoperation compared to one another.

## Methods

### Study methodology and procedures

We conducted an observational study of 200 tibial shaft fractures to determine what fracture characteristics are predictive of a nonunion and the need for a reoperation. Medical records (clinical notes and radiographs) of patients with tibial shaft fractures were reviewed. This study was approved by the Research Ethics Board (project number 10-595-C under the Hamilton Health Sciences/McMaster Health Sciences, Research Ethics Board).

### Patient eligibility

Consecutive patients with tibial shaft fractures were identified at three sites within a university-affiliated teaching hospital. Patients were identified through their hospital medical records by the Health Records Department. The study included patients who were treated for acute fractures and excluded those that were referred for a complication. In addition, patients were required to fulfill the following eligibility criteria: (1) 19 years of age or older, (2) tibial fracture requiring operative fixation with internal fixation, (3) sufficient clinical information available within the patient’s medical record and (4) radiographs available for the assessment. Tibial fractures that extended into the joint, bilateral fractures and Gustilo-Anderson Type IIIc open fractures were excluded. Two individuals independently assessed each identified patient’s electronic medical records to determine if they met the pre-defined eligibility for this study. Any disagreements were resolved by a third individual and reasons for exclusion were documented.

### Definitions

Reoperation was defined as any invasive procedure completed to promote fracture healing. Such invasive measures included, but were not limited to, bone grafting, nail dynamization, or implant exchange. Nonunions were determined by the patient’s attending physician as dictated in their clinical records and identified during the data collection process.

### Identification of prognostic variables

Study specific case report forms were developed for this study. The case report forms were developed using previous literature and 15 surgeons in the field of orthopaedic trauma surgery. A sampling to redundancy strategy was utilized, by which additional experts were contacted until no new items were generated for the case report forms. A panel of experts rated their perceived importance of each identified factor on a scale of one to ten, with ten as the top priority to include in the analysis and one as the lowest priority to include in the analysis. The highest ranked factors were therefore included on the case report forms. The case report forms were piloted on ten sample patients before the study was initiated. The pilot test data was reviewed to confirm that the case report forms captured the information it was intending to collect.

### Data collection

Two individuals independently extracted the data from each patient’s electronic medical record. The two individuals reviewed all available medical records including initial hospital notes from the emergency room consultation, the surgical consultation, the operative report(s), in-hospital progress reports, the hospital discharge summary, all fracture clinic notes, subsequent operative report(s) (if applicable), and all re-hospitalization notes (if applicable) related to the patient’s included fracture. They recorded the data on the study-specific case report forms. Any disagreements were resolved by a third individual, who independently extracted the data in which there was a discrepancy.

### Radiographic evaluation

An independent reviewer assessed the initial x-ray, the post-operative x-ray, and all available sequential x-rays. The independent reviewer assessed the radiographs and documented fracture characteristics, post-operative characteristics and any identified radiographic complications on the case report forms. Fracture characteristics included location of fracture, type of fracture, cortical continuity and bone loss. Post-operative characteristics included size of fracture gap and amount of cortical continuity (lack of bridging of tibial cortices), which were used as indicators of healing.

### Data management

Data from both the available patient medical records and data from the case report forms completed during radiographic evaluation were consolidated and, in preparation for analysis, entered into a database and a subset of the data was reviewed. Reporting adverse events was not applicable, as the data sources used in this study did not contain physician attribution of causality of adverse events to any medicinal or surgical products.

### Sample size determination

Our previous randomized trial in patients with tibial shaft fractures identified an event rate of a secondary intervention of 14% (95% CI: 12-16%) in closed fractures and 27% (95% CI: 22-31%) in open fractures [26]. Given a sample size of 200 patients with tibial shaft fractures treated operatively, we anticipated to have 50 patients (25%) with one healing complication leading to an expected outcome event per predictor variable (EPV) of 10 for the logistic regression model. Based on simulation studies, EPV values of 10 (or greater) showed no major issues in terms of confidence interval coverage, type I error, relative bias and other model performance measures [27,28].

### Statistical analysis

Data was analyzed using SPSS Version 21.0 [SPSS, Chicago, IL]. Our primary analyses included looking at nonunions and reoperations.

Dichotomous data was reported as number of participants and proportions, with corresponding confidence intervals to estimate precision. Continuous data was presented as means and medians with standard deviations. Descriptive statistics describing the patients’ demographics, fracture characteristics, fracture complications and management of the documented fracture complications were also provided.

The primary analysis was a multivariable logistic regression using the primary outcome (need for reoperation) as the dependent variable looking for predictor variables in patients with tibial shaft fractures at baseline. The same analysis was completed to assess fracture characteristics and their predictive value using nonunion as the secondary outcome.

We also conducted an exploratory regression analysis comparing different aggregates of two of the three fracture characteristics included in a previously-developed prognostic index for their predictive value using reoperation and nonunion as outcomes.

A chi-square test was performed to test the null hypothesis of no association between nonunion and reoperation.

## Results

### Characteristics of the patients

We identified 594 potentially eligible patients from the hospital database review. Of these, 200 were included in our analysis. Patients were excluded if they had an insufficient amount of follow-up clinical or radiographical data (141), a tibial fracture not fixed via operative internal fixation (108), a fracture extending into the joint (87), a non-tibial fracture (35), a bilateral fracture (17), or were under the age of 18 (6). The typical patient was a healthy male (69.0%), averaging 42 years of age, who consumed alcohol (73%), and had no previous injuries to the ipsilateral tibia (98%) (Table 1). Most tibial fracture injuries were accompanied by injuries to the ipsilateral fibula (92%). Of the fractures included, 43% were a result of a motor vehicle-related incident. Fracture injuries tended to be closed (78%) and were treated with reamed intramedullary nailing (81%). Post-operatively, patients did not suffer from any fracture complications during their hospital stay (97%), which was often less than a week (Table 2).

**Table 1:** Characteristics of the patients

**Characteristic**	**No. (%) of subjects**	
*Group sample size*	N = 200	
*Gender (n = 200)*		
Male	138 (69.0)	
Female	62 (31.0)	
*Age (n = 200)*		
Mean age in years	42 ±16.5	
Diabetes (n =191)*		
Type II (Insulin dependent diabetes)	14 x(7.3)	
Type I (Non-insulin dependent diabetes)	10 (5.2)	
No	167 (87.4)	
Tobacco use *(n = 138)*		
Yes	64 (45.7)	
No	60 (43.5)	
Previously used	15 (10.9)	
*Health history (n =189)*		
None	137 (72.5)	
Vascular disease	46 (24.3)	
Myocardial Infarction and vascular disease	3 (1.6)	
Stroke and vascular disease	2 (1.1)	
Myocardial infarction	1 (0.5)	
*Alcohol use (n = 95)*		
Yes	69 (72.6)	
No	26 (27.4)	
*Steroid use (n = 170)*		
Yes	14 (8.2)	
No	156 (91.8)	
*Previous injuries to the ipsilateral tibia (n = 183)*		
Yes	4 (2.2)	
No	179 (97.8)	

*n denominators do not equate to 200 due to missing values.

**Table 2:** Fracture characteristics

**Characteristic**	**No. (%) of subjects**	
*Other injuries (n = 200)*		
Yes	183 (91.5)	
No	17 (8.5)	
*Side of injury (n =200)*		
Right	112 (56.3)	
Left	87 (43.7)	
Mechanism of injury (n = 197)*		
Fall from standing	43 (21.8)	
Motor vehicle accident	34 (17.3)	
Twist	26 (13.2)	
Pedestrian motor vehicle accident	22 (11.2)	
Motorcycle accident	19 (9.6)	
Crush	18 (9.1)	
Recreational vehicle accident	9 (4.6)	
Fall from height	8 (4.1)	
Other	18 (9.1)	
*Degree of soft tissue injury (n =165)*		
Closed	129 (78.2)	
Open type 1	12 (7.3)	
Open type 2	16 (9.7)	
Open type 3A	4 (2.4)	
Open type 3B	4 (2.4)	
*Method of fixation (n =193)*		
Reamed IM nailing	156 (80.8)	
Plate fixation	36 (18.7)	
Unreamed IM nailing	1 (0.5)	
*Post operative weightbearing status (n = 100)*		
Nonweightbearing	61 (61.0)	
Partial weightbearing	38 (38.0)	
Full weightbearing	1 (1.0)	
*Hospital stay post surgery (n = 199)*		
*Mean stay in days*	9.5 ±16.3	
*Fracture complication during hospital stay (n = 144)*		
No	139 (96.5)	
Yes	5 (3.5)	

*n denominators do not equate to 200 due to missing values.

### Nonunions and reoperations

In our cohort of tibial fractures, 37 (18.5%) went on to nonunion, as identified by the attending physician. Of the 37 patients with nonunion, 62.2% of them were identified within the first six months of the initial injury. Additionally, 27 (13.5%) of all included patients underwent a reoperation, with 55.6% of them occurring within the first six months of the initial injury.

### Factors associated with nonunion and reoperation

Univariable analysis identified six fracture characteristics associated with the incidence of nonunion (p < 0.05), which included fractures with less than 25% cortical continuity (odds ratio = 6.44 [95% CI 1.89, 21.95]; p = 0.003), open fractures (odds ratio = 2.56 [95% CI 1.24, 5.29]; p = 0.011), the presence of comminution (odds ratio = 2.21 [95% CI 1.05, 4.66]; p = 0.037), and an oblique (odds ratio = 2.94 [95% CI 1.32, 6.58]; p = 0.009) or segmental (odds ratio = 3.17 [95% CI 0.96, 10.46]; p = 0.058) fracture type (Table 3). Multivariable logistic regression analysis suggested that only cortical continuity remained predictive (odds ratio = 4.72 [95% CI 1.33, 16.76]; p = 0.02) (Table 4).

**Table 3:** Univariable logistic regression model for predictors of nonunion

**Factor**	Group sample size*	**Nagelkerke R square**	**Odds ratio**	
[95% CI]	Probability			
*Cortical Continuity*	193	0.108	6.444 [1.892, 21.950]	0.003
50-100%				
0-25%				
*Degree of Soft Tissue Injury*	199	0.520	2.561 [1.238, 5.295]	0.011
Closed				
Open				
*Comminution*	194	0.035	2.210 [1.048, 4.660]	0.037
No				
Yes				
*Transverse Fracture*	199	0.011	1.897 [0.682, 5.276]	0.220
No				
Yes				
*Oblique Fracture*	190	0.070	2.942 [1.316, 6.579]	0.009
Transverse				
Oblique				
*Segmental Fracture*			3.173 [0.963, 10.459]	0.058
Transverse				
Segmental				

*n denominators do not equate to 200 due to missing values.

**Table 4:** Multivariable logistic regression model for predictors of nonunion

**Factor**	**Odds ratio [95% CI]**	**Probability**
Group Sample Size* =189		
*Nagelkerke R Square = 0.172*		
*Cortical Continuity*	4.716 [1.327, 16.761]	0.017
50-100%		
0-25%		
*Degree of Soft Tissue Injury*	1.262 [0.533, 2.985]	0.597
Closed		
Open		
*Comminution*	0.989 [0.398, 2.457]	0.981
No		
Yes		
*Oblique Fracture*	2.064 [0.832, 5.116]	0.118
Transverse		
Oblique		
*Segmental Fracture*	1.957 [0.510, 7.505]	0.328
Transverse		
Segmental		

*n denominators do not equate to 200 due to missing values.

Alternatively, open fractures and transverse fractures were the only fracture characteristics that showed any significant predictive value for reoperation, as shown via univariable analysis (Table 5). All tibial fractures with less than 25% cortical continuity accounted for all 27 reoperations we identified in our sample. The presence of a transverse fracture was the only variable to approach significance for the incidence of reoperation in the multivariable logistic regression analysis (odds ratio = 3.03 [95% CI 1.00, 9.18]; p = 0.05) (Table 6).

**Table 5:** Univariable logistic regression model for predictors of reoperation

**Factor**	Group sample size*	**Nagelkerke R square**	**Odds ratio [95% CI]**	**Probability**
*Degree of Soft Tissue Injury*	199	0.065	3.094 [1.347, 7.109]	0.008
Closed				
Open				
*Comminution*	194	0.006	1.417 [0.606, 3.311]	0.422
No				
Yes				
*Transverse Fracture*	199	0.034	2.990 [1.046, 8.551]	0.041
No				
Yes				
*Oblique Fracture*			2.020 [0.820, 4.978]	0.127
Transverse				
Oblique				
*Segmental Fracture*	190	0.034	2.825 [0.784, 10.180]	0.112
Transverse				
Segmental				

*n denominators do not equate to 200 due to missing values.

**Table 6:** Multivariable logistic regression model for predictors of reoperation

**Factor**	**Odds ratio [95% CI]**	**Probability**
Group Sample Size* =194		
*Nagelkerke R Square = 0.152*		
*Degree of Soft Tissue Injury*	1.993 [0.782, 5.079]	0.149
Closed		
Open		
*Transverse Fracture*	3.027 [0.998, 9.178]	0.050
No		
Yes		

*n denominators do not equate to 200 due to missing values.

### Secondary treatment profile for nonunions

Of the 37 nonunions reported in this study, 32.4% of them were treated with a noninvasive therapy option alone to promote bony union. Such options include, but are not limited to, ultrasound therapy, electrical stimulation, or medication. Additionally, 40.5% of the nonunions were treated with a reoperation alone and 24.3% were treated with a combination of both noninvasive therapies and a reoperation. There was only one case who did not receive any secondary treatment for nonunion.

### Validation of previous prognostic index for reoperation

A previous index identified open fractures, presence of a fracture gap post-fixation, and a transverse fracture type as variables in a predictive model for reoperation within one year following operative management of tibial fractures [25].

Using the same prognostic risk model (Table 7), our findings largely confirmed the incremental increase in the risk of reoperation with one, two, and three prognostic risk factors. Patients with at least two of the three risk factors were more likely to develop a nonunion, with the presence of both a fracture gap and open fracture having the highest predictive value for a nonunion over any other aggregate of two of the three variables included in the prognostic risk model (Table 8). Patients with at least two or all three risk factors were more likely to incur a reoperation. All possible aggregates of two of the three prognostic risk variables provided a predictive basis for reoperation (Table 9).

**Table 7:** Risk of reoperation

**Condition**	**Previous study**	**Current study**
	**(2003)**^**25**^	**(2013)**
	N = 192	N = 193
No Risk Factors*	3.8%	0.0%
One Risk Factor	17.7%	11.7%
Two Risk Factors	47.0%	23.7%
Three Risk Factors	94.0%	44.4%

*Risk factors include: Fracture gap, Open fracture, Transverse fracture.

**Table 8:** Logistic regression model for predictors of nonunion using a set and combination of three previously-identified prognostic variables

**Factor**	Group sample size*	**Nagelkerke R square**	**Odds ratio [95% CI]**	**Probability**
*One Risk Factor*^*±*^	193	0.007	0.708 [0.330, 1.517]	0.374
*Two Risk Factors*	193	0.045	2.450 [1.165, 5.154]	0.018
*Three Risk Factors*	193	0.028	3.800 [0.967, 14.938]	0.056
*Combination of Two Risk Factors*				
Fracture Gap and Open Fracture	193	0.083	3.382 [1.601, 7.143]	0.001
Fracture Gap and Transverse Fracture	193	0.020	2.417 [0.841, 6.947]	0.101
Open Fracture and Transverse Fracture	199	0.021	3.152 [0.842, 11.794]	0.088

*n denominators do not equate to 200 due to missing values.

^±^Risk factors include: Fracture gap, Open fracture, Transverse fracture.

**Table 9:** Logistic regression model for predictors of reoperation using a set and combination of three previously-identified prognostic variables

**Factor**	Group sample size*	**Nagelkerke R square**	**Odds ratio [95% CI]**	**Probability**
*One Risk Factor*^*±*^	193	0.005	0.721 [0.306, 1.699]	0.454
*Two Risk Factors*	193	0.057	2.896 [1.264, 6.633]	0.012
*Three Risk Factors*	193	0.048	5.600 [1.401, 22.382]	0.015
*Combination of Two Risk Factors*				
Fracture Gap and Open Fracture	193	0.106	4.294 [1.847, 9.984]	0.001
Fracture Gap and Transverse Fracture	193	0.045	3.667 [1.244, 10.806]	0.018
Open Fracture and Transverse Fracture	199	0.042	4.812 [1.262, 18.343]	0.021

*n denominators do not equate to 200 due to missing values.

^±^Risk factors include: Fracture gap, Open fracture, Transverse fracture.

### Association between nonunions and reoperations

Chi-square analysis found an association between nonunion and reoperation, χ^2^ (1, N = 198) = 101.4, p < 0.001. 12.1% of all included patients experienced both a nonunion and a reoperation and patients with a nonunion were 97 times (95% CI 25.8-366.5) more likely to have a reoperation.

## Discussion

### Key findings

Our multivariate regression analysis further corroborates the well-supported concept that a lack of cortical continuity (<25%) is a strong predictor of nonunion and reoperation [6,11,25,29,30]. Although the presence of an open fracture was not a significant covariate in the multiple logistic regression model, open fractures are known prognostic factors for complications following fracture fixation [31-35] and this characteristic demonstrated a significant association to the incidence of both nonunion and reoperation in the univariate models, suggesting a possible study power issue. Our findings suggest a trend towards increased reoperations with transverse fractures compared to oblique or spiral fractures and is consistent with previous reports of their characteristic association to high-impact injuries and the need for reoperation to achieve bony union in such cases [11,25].

Using our cohort of patients, we were able to independently evaluate the validity of a previous prognostic index. This index used three variables (open fracture, presence of a fracture gap post-fixation, and transverse fracture type) and was developed using a cohort of 200 patients [25]. Using our study patients as a validation cohort, we are able to see the increase in reoperation rates in patients who satisfied the prognostic variable criterion, thereby confirming the predictive value of the risk index.

Logistic regression analysis using different aggregates of two of the three previously-developed prognostic risk variables indicated that a grouping of both a fracture gap and open fracture together was the only significant predictor for the incidence of nonunion. Using this observation to build upon the predictive value of the previously-identified risk index, such patients can be considered as having fractures of high risk for the development of nonunion and can therefore be more accurately identified to facilitate treatment options more appropriate for achieving bony healing.

It is of interest to note that although only a combination of a fracture gap and an open fracture was found to be significantly attributed to the incidence of nonunion alone, all combinations of the three previously-identified prognostic variables demonstrate predictive value for reoperation. The results indicate that although all included patients who experienced a reoperation had a tibial fracture with less than 25% cortical continuity, not all patients with a diagnosed nonunion, as determined by their attending physician, satisfied this same criterion. This observation leads us to believe that all reported reoperations in this study were performed in an effort to achieve bony union in a fracture showing little to no progression towards healing on its own [36]. There is potential evidence here that surgeons may be performing reoperations on fracture patients with the expectation of developing nonunion rather than the incidence of it. There is still a general consensus across the orthopaedic community that prognostic variables predicting fracture complications such as nonunion or delayed union remain poorly defined [4] and if nonunions are indeed being treated prematurely, further studies on the relationship between reoperations and the proceeding development of fracture union could be warranted based on these findings to truly determine the therapeutic effect of a reoperation and prevent unnecessary and potentially problematic secondary surgery for fracture patients.

In addition to the validation of a previously-developed prognostic risk index for reoperation, we have identified three important findings: (1) tibial fractures with less than 25% cortical continuity have the highest predictive value of nonunion and reoperation than any other fracture characteristic included in this study, (2) the combination of a fracture gap post-fixation and an open fracture yields patients that are at high risk for developing nonunion (3) orthopaedic surgeons may be prematurely treating patients with anticipated nonunion rather than the incidence of it via invasive surgical methods.

### Strengths and limitations

Our findings are strengthened by (1) involving a number of experts during development of the case report forms and prognostic factor list; (2) using a comprehensive sampling of patients from three hospital sites within three university-affiliated academic centers; (3) including only patients with sufficient amount of follow-up data to identify healing or the occurrence of a bone healing complication; (4) choosing an outcome measure that is objective and of unequivocal importance to patients; (5) our thorough data collection and analytic methods, which included a compilation of data extracted from patient charts (completed independently by two reviewers) and separately from the radiographs.

The results have limitations, which include (1) our sample may not be completely representative of all tibial fracture patients, but only reflects those treated within three university affiliated academic centers; (2) the identified prognostic factors may only be generalizable to tibial fracture outcomes; (3) as a retrospective review, the data collected from patient charts may not always be reliable or complete; (4) our sample size may have been insufficient to definitively power our regression analyses.

## Conclusions

The evidence from this study has identified a significant association between cortical continuity and both nonunions and the need for reoperation in tibial shaft fractures. In addition, our study reconfirmed the prognostic risk index proposed from an earlier study, which listed the presence of a fracture gap post-fixation, open fractures and transverse fracture types as risk factors for nonunion and reoperations in tibial shaft fractures. While severity of soft tissue injury and fracture type remain non-modifiable risk factors, cortical continuity, in many cases can be modified and better monitored via additional clinical assessment tools such as computed tomography (CT) supplementary and alternative to radiographic examination. Surgeon attention to technical aspects of the surgical procedure to obtain apposition of fracture ends and avoid gaps is paramount. Clinical assessment of the effectiveness of invasive surgery for achieving bony union can help determine if currently published prognostic risk factors for nonunion requiring reoperation are accurate, as our study provides evidence that surgeons may be performing additional surgery to treat the anticipation of nonunion development. Regardless, surgeons can use this information to guide discussions about patient prognosis following operative management of tibial fractures.

## Competing interests

Funding for this research was provided by AMGEN Inc. Dr. Mohit Bhandari was funded, in part, by a Canada Research Chair.

## Authors’ contributions

Study Conception and Design: MB, SS. Acquisition of Data: MB, SS, KF, VT, CJF. Analysis and Interpretation of Data: MB, SS, KF. Drafting of the Manuscript: MB, SS, KF, VT. Critical Revision of the Manuscript: MB, CJF, BP, DW, BR, SS. Statistical Expertise: MB. Administrative, Technical, or Material Support: MB, SS. Study Supervision: MB, SS. All authors read and approved the final manuscript.

## Pre-publication history

The pre-publication history for this paper can be accessed here:

http://www.biomedcentral.com/1471-2474/14/103/prepub
